# Targeted NMDA receptor knockdown in recall‐activated neuronal ensembles impairs remote fear extinction

**DOI:** 10.1186/s13041-025-01203-z

**Published:** 2025-04-05

**Authors:** Yongmin Sung, Dae Hee Han, Junhyuk Kim, Pojeong Park, Bong-Kiun Kaang

**Affiliations:** 1 Center for Cognition and Sociality, Institute for Basic Science (IBS), Daejeon, 34141 South Korea; 2 Department of Biological Sciences, College of Natural Sciences, Seoul National University, Seoul, 08826 South Korea; 3 Interdisciplinary Program in Neuroscience, Seoul National University, Seoul, 08826 South Korea; 4 Department of Brain Sciences, DGIST, Daegu, 42988 South Korea

**Keywords:** Remote fear memory extinction, Recall-activated neurons, N-methyl-d-aspartate receptors, Clustered regularly interspaced short palindromic repeats (CRISPR)

## Abstract

Fear extinction training in rodents decreases fear responses, providing a model for the development of post-traumatic stress disorder therapeutics. Fear memory recall reactivates the consolidated fear memory trace across multiple brain regions, and several studies have suggested that these recall-activated neurons are re-engaged during extinction. However, the molecular mechanisms linking this reactivation to extinction remain largely elusive. Here, we investigated the role of N-Methyl-d-Aspartate receptors (NMDARs) in remote memory recall–activated neurons within the basolateral amygdala and the medial prefrontal cortex during extinction training in mice. We found that *Grin1* knockdown in these specific ensembles impaired extinction of remote fear memory, but did not reduce their reactivation during retrieval of the extinguished memory. These data suggest that while reactivation of these neuronal populations persists, their NMDARs are crucial for driving the synaptic plasticity needed to extinguish remote fear memories.

**Supplementary Information:**

The online version contains supplementary material available at 10.1186/s13041-025-01203-z.

## Main text

Extinction of obsolete fear memories is essential for effective adaptation to new environments. Rodent studies have provided both correlational [1] and causal [2, 3] evidence that neuronal activity in the infralimbic region of the medial prefrontal cortex (mPFC) and the basolateral amygdala (BLA) is crucial for successful extinction training. Furthermore, the activities of reciprocal connections between these regions underlie the extinction [4].

Recently, the reactivation of remote recall-activated neuronal ensembles has been suggested as a cellular mechanism of remote fear memory extinction [5]. However, the molecular mechanisms by which these reactivated neurons contribute to the attenuation of fear memory remain elusive. Given the pivotal role of N-methyl-d-aspartate receptors (NMDARs) in the BLA and the mPFC in extinction [6, 7], we hypothesized that NMDARs of the recall-activated neuronal ensembles function as a critical mediator of fear memory extinction.

To test whether NMDARs in remote recall-activated neurons in the BLA and the mPFC are necessary for the extinction training, we induced knockdown (KD) of NMDARs in these neurons by combining an activity-dependent labeling system with a virally delivered cre-dependent CRISPR-Cas9 system. A cocktail of AAVs encoding cre-dependent tdTomato and SaCas9 cassette was bilaterally infused to the BLA and the mPFC of Arc-CreERT2 mice. The SaCas9 cassette contained either sgRNA targeting exon 1 of the *Grin1* gene (sgGrin1) or control sgRNA (3-base pair mismatch at the 3′ end, sgGrin1 ATG) (Supplementary Fig. 1 A). In a parallel experiment, we confirmed that both CaMKII-driven SaCas9 expression delivered via the AAV system and SpCas9 expression in a knock-in mouse significantly decreased the NMDA/AMPA ratio (Supplementary Fig. 1B–E).

**Fig. 1:**
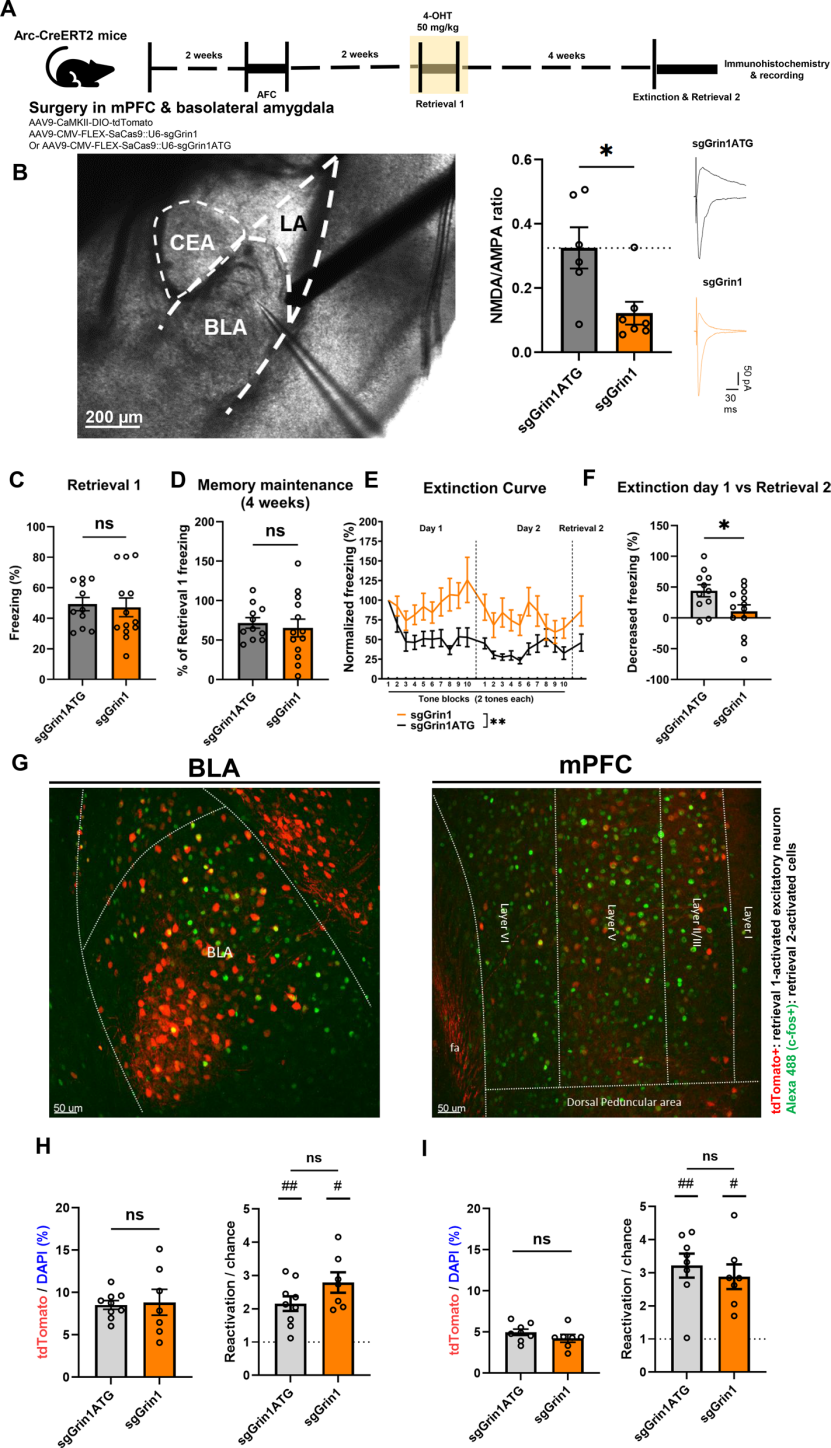
NMDAR KD in the recall-activated ensembles in the BLA and the mPFC. **A** Behavioral experiment scheme. 4-OHT was delivered two hours before remote retrieval 1, to tag ensembles activated during retrieval 1. **B** Left, a representative image of the recorded slice. Middle, sgGrin1 group showed a significantly lower level of NMDA/AMPA ratio than the sgGrin1 ATG group. Each dot represents a cell; grey, control group injected with scrambled sgRNA (n = 6 cells); orange, test group injected with sgGrin1 (n = 7 cells). Right, a representative EPSC trace of each group. Mann–Whitney test, *p = 0.0350. **C** Freezing level during retrieval 1. Unpaired *t*-test, ns, p = 0.7884. **D** Retention of fear memory after 4-OHT induction measured by the first tone block of the extinction curve. sgGrin1 ATG group, N = 13; sgGrin1 group, N = 11. Unpaired *t*-test, ns, p = 0.6485. **E** Trace of the freezing level during the extinction day 1 and 2. Each tone block represents the average freezing level of consecutive two tones. sgGrin1 ATG group, N = 13; sgGrin1 group, N = 11. Two-way repeated measures ANOVA; time effect, p = 0.0923; *Grin1* KO effect, **p = 0.0068; **F** Extinction index of the sgGrin1 group and the sgGrin1 ATG groups. sgGrin1 ATG group, N = 13; sgGrin1 group, N = 11. Mann–Whitney test, *p = 0.0410. **G** Representative image of c-fos immunohistochemistry analysis. **H** Left panel, the proportion of tdT + neurons among DAPI + cells in the BLA. Each dot represents the average value of images from an individual mouse. sgGrin1 ATG group, N = 9; sgGrin1 group, N = 7; Mann–Whitney test, ns, p = 0.9182; right panel, Reactivation/chance = P(cfos|tdTomato)/P(cfos|DAPI); Mann–Whitney test, ns, p = 0.2105; One-sample Wilcoxon test, #p = 0.0156, ##p = 0.0039. **I** Left panel, the proportion of tdT + neurons among DAPI + cells in the mPFC. Each dot represents the average value of images from an individual mouse. sgGrin1 ATG group, N = 8; sgGrin1 group, N = 7; Mann–Whitney test, ns, p = 0.1893; right panel, Reactivation/chance = P(cfos|tdTomato)/P(cfos|DAPI); Mann–Whitney test, ns, p = 0.3357; One-sample Wilcoxon test, #p = 0.0156, ##p = 0.0078. Data are presented as mean ± SEM

Two weeks after auditory fear conditioning, mice underwent remote recall (Retrieval 1) of auditory fear memory during which 4-hydroxytamoxifen (4-OHT; 50 mg/kg) was administered to capture recall-activated ensembles for the *Grin1* KD via CRISPR-Cas9 system (Fig. 1A). Four weeks later, mice were subjected to 2 days of extinction training and the next day, mice were sacrificed after a memory test (Retrieval 2) for post hoc electrophysiological analysis and immunohistology. In the BLA, tdTomato-expressing (tdT+) neurons from the sgGrin1 group exhibited significantly decreased levels of NMDAR-mediated currents compared to counterparts from the sgGrin1 ATG group indicating successful KD of NMDARs in recall-activated neurons (Fig. 1B). Immunohistochemical analysis revealed the significant colocalization of the Cas9 and the tdTomato proteins, and decreased level of GluN1 in tdT + neurons compared to surrounding tdT- neurons, further validating the specificity of our KD system (Supplementary Fig. 2 A–D).

The freezing levels were comparable between the sgGrin1 and the control group during Retrieval 1 and four weeks after Retrieval 1, indicating that memory retention was not impaired by *Grin1* KD (Fig. 1C, D). However, the *Grin1* KD group showed a significantly disrupted extinction curve (Fig. 1E) and an abolished effect of extinction at Retrieval 2 (Fig. 1F). Nevertheless, tdT + neurons in both the mPFC and the BLA remained significantly responsive compared to chance levels, regardless of *Grin1* KD (Fig. 1G–I).

In this study, we found that NMDARs in remote recall-activated ensembles within the BLA and the mPFC are crucial for the extinction of remote fear memory. Notably, despite the *Grin1* KD, these neuronal ensembles in the BLA and the mPFC remained responsive to the CS during the recall after extinction.

It was hypothesized that extinction-induced reactivation of previous memory traces may provide a window for updating maladaptive fear memories [8]. In line with this notion, reactivation of neuronal ensembles in the dentate gyrus tagged during remote memory recall was necessary for extinction [5]. However, the molecular mechanisms linking such reactivation to the process of fear extinction remain unclear.

Previous studies utilizing region-specific conditional KD [9] or infusion of NMDAR antagonists [3, 10] demonstrated the critical role of NMDARs in extinction. While NMDAR blockade in the BLA impaired within-session extinction, the same approach in the infralimbic cortex (IL) led to the failure of extinction memory consolidation. In particular, the effectiveness of extinction training was correlated with NMDAR-dependent burst firing in IL neurons [10, 11] and extinction training-induced plasticity of NMDAR-mediated currents at ventral hippocampal synapses in the IL [12]. However, these earlier approaches lacked the cellular specificity needed to pinpoint how particular neuronal ensembles contribute to extinction learning. By combining activity-dependent tagging with target gene KD, we were able to investigate the critical role of NMDARs specifically within the remote recall-activated ensembles of the BLA and the mPFC.

Our results align with and extend previous findings in several key ways. First, the *Grin1* KD in the recall-activated neurons in the BLA and the mPFC did not affect memory retention. To note, prolonged knockout of *Grin1* in the forebrain significantly impaired remote fear memory retention [9]. This discrepancy may stem from the brain-wide nature of the memory trace [13], allowing other regions to compensate for local *Grin1* KD in the BLA and mPFC. Second, we found a significantly impaired extinction of remote fear memory by *Grin1* KD in recall-activated neurons. Indeed, earlier studies showed that the blockade of NMDARs in the mPFC [10] or BLA [7] impaired fear memory extinction. Our findings further suggest that the essential NMDARs identified in those experiments may specifically reside in the recall-activated ensembles.

Our results indicate that NMDARs in recall-activated neurons are required for fear memory extinction but are not essential for their reactivation during fear memory recall. Therefore, it is unlikely that NMDARs in these neurons mediate the unlearning of the fear memory trace through an LTD-like process at their synaptic inputs [14]. Instead, they may facilitate new learning of safety signals that suppress the previously established fear memory [15]. One possibility is that NMDARs enhance burst firing in recall-activated neurons, thereby promoting synaptic plasticity in downstream regions when combined with convergent inputs [10].

In summary, by incorporating the activity-dependent tagging system and the AAV-delivered CRISPR-Cas9 system, we showed that NMDARs in recall-activated ensembles in the BLA and mPFC are required for the extinction of fear memory. These findings provide important insight into how recall of fear memory is linked to extinction.

## Methods

### Mice

All experiments were performed using 8–16-week-old C57BL6/N (Samtako.Bio. Korea), ArcCreER^T2^ (ArcTRAP; Jackson Labs; stock #021881) and LSL-Cas9 (Jackson labs; stock #026175). Mice were raised in a 12-h light/dark cycle in standard laboratory cages and given ad libitum access to food and water. All procedures and animal care followed the regulations and guidelines of the Institutional Animal Care and Use Committees (IACUC) of Seoul National University or IBS (Daejeon, Korea).

### DNA constructs

pAAV-FLEX-SaCas9-U6-sgRNA (Addgene plasmid # 124844; http://n2t.net/addgene:124844; RRID: Addgene_124844) and pAAV-FLEX-SaCas9-U6-sgGrin1(Addgene plasmid # 124852; http://n2t.net/addgene:124852; RRID:Addgene_124852) was a gift from Larry Zweifel. For the control sgRNA-containing cassette, the annealed oligos were inserted into the FLEX-SaCas9-U6-sgRNA plasmid following the author’s instruction [16].

### Adeno-associated virus production

Briefly, plasmid containing the construct of interest flanked by AAV2 ITR, pAd-ΔF6, pRep/Cap9 were co-transfected into AAVpro® 293 T Cell Line (TAKARA, cat# 632273) and incubated in DMEM 10% v/v FBS for 5 days in 150 mm culture dish. On the harvest day, viruses in the culture medium were collected and remaining cells were broken to extract virus within it. After the centrifugation at 13,490×*g* for 20 min at 4 °C, supernatants were carefully loaded to the ultracentrifugation tubes filled with iodixanol gradient solutions as previously described (Fripont, 2019, Jove). 40% iodixanol solutions were aspirated using syringes and filtered by pre-rinsed centrifugal filters (Merck, cat# UFC910024).

### Auditory fear conditioning

All mice were fear-conditioned 2–3 weeks after the AAV injection. Each mouse was habituated to the hands of the investigator and anesthesia chamber without isoflurane for 7 consecutive days. In all experiments, fear conditioning and extinction occurred in two different contexts (context A and context B) to minimize the influence of contextual associations. Context A consists of a square chamber with a steel grid floor (Coulbourn instruments; H10 - 11 M-TC), and context B consists of a rectangular plastic box with striped walls and with a hardwood laboratory bedding (betachip). 2 h before the conditioning, 250 μl of 5 mg/ml Doxycycline solution dissolved in saline was injected intraperitoneally during brief anesthesia by isoflurane. For auditory fear conditioning, mice were placed in context A and allowed to explore the context for 150 s, followed by three exposures to auditory tone CS (30 s), each of which co-terminated with 2 s, 0.75 mA footshock US, with a 30 s inter-trial interval (Lim et al., 2017). After the conditioning, mice were immediately delivered to their home cages. 1 day after the conditioning, mice were placed into a novel context B and exposed to the auditory tone to measure the freezing behavior. The freezing behavior was recorded and scored using a FreezeFrame fear-conditioning system.

### Fear extinction

Four weeks after the auditory fear conditioning, all groups of mice underwent extinction. For two consecutive days, mice in the extinction group were placed into context B. After a 2-min exploration period, the auditory tone was administered 20 times with a 30-s inter-trial interval in the absence of the footshock. One day after the last extinction session, mice were placed into context B and exposed to the auditory CS to measure the freezing behavior. The extinction index was calculated by (Freezing of 1 st tone block of Extinction day 1 − Freezing of Retrieval 2)/(Freezing of 1 st tone block of Extinction day 1). Only animals with confirmed virus expression in both the BLA and the mPFC were included in the analysis.

### Stereotaxic surgery

Mice were anesthetized with a ketamine/xylazine solution and positioned on a stereotaxic apparatus (Stoelting Co. Cat. No. 51733 or RWD). The virus mixture was injected into target regions using a 32-gauge needle with a Hamilton syringe at a rate of 0.125 μl/min. The total injection volume per site was 0.5 μl, and the tip of the needle was positioned 0.05 mm below the target coordinate right before the injection for 2 min. After the injection was completed, the needle stayed in place for an extra 6 min and was withdrawn slowly. Stereotaxic coordinates for each target site were: basolateral amygdala (AP: − 1.4/ML: ± 3.4/DV: − 5.05), medial prefrontal cortex (AP: + 1.8/ML: ± 0.4/DV: − 2.75), hippocampal CA1 (AP: + 1.75/ML: ± 1.4/DV: − 1.65 from dura).

### Sample preparation and confocal imaging

Perfused brains were fixed with 4% paraformaldehyde in phosphate buffered saline (PBS) overnight at 4 °C, and dehydrated in 30% sucrose in PBS for 2 days at 4 °C. Brains were sliced by Cryostat into a 40 μm section for immunohistochemistry. The BLA and the mPFC were imaged in a Z stack using a Leica SP8 confocal microscope with a 20× objective lens.

### Electrophysiology

Mice were deeply anesthetized with ketamine and checked for tail-pinching reflex. Animals were then transcardially perfused with ice-cold sucrose-ACSF that contained (in mM): 210 sucrose, 3 KCl, 26 NaHCO_3_, 1.25 NaH_2_PO_4_, 5 MgSO_4_, 10 D-glucose, 3 sodium ascorbate and 0.5 CaCl_2_, saturated with 95% O_2_ and 5% CO_2_ (pH adjusted to 7.35 with HCl, 300–310 mOsm). Subsequently, the brain was removed and allowed to chill in the solution ~ 30 s. After mounting the brain on the agar block (3% w/v), slices were obtained using a vibratome (VT1200S, Leica) [17]. Transverse hippocampal slices or coronal slices containing BLA or mPFC were prepared and immediately transferred to normal-ACSF at 32–34 °C and allowed to recover there for 30 min. Normal ACSF contained (in mM): 124 NaCl, 3 KCl, 26 NaHCO_3_, 1.25 NaH_2_PO_4_, 2 MgSO_4_, 15 D-glucose and 1 or 2 CaCl_2_ (carbonated with 95% O_2_ and 5% CO_2_). Subsequently, slices were moved and allowed to recover at room temperature at least for 1 h before the recordings were made.

NMDA/AMPA ratio was measured using whole-cell solution that contained (in mM): 8 NaCl, 130 CsMeSO_3_, 10 HEPES, 0.5 EGTA, 4 Mg-ATP, 0.3 Na3-GTP, 5 QX- 314 and 0.1 spermine. Whole-cell recording was performed at 32 °C during continuous perfusion at 3–4 ml/min with ACSF that contained 100 μM picrotoxin. After 5 min of baseline recording, consecutive 10 responses to evoked EPSCs were measured in − 70 mV holding potential for AMPAR-currents. NMDAR-currents were estimated at 50 ms after the stimulation onset at + 40 mV of holding potential.

### Immunohistochemistry

45 μm sections were rinsed three times in 1 × PBS. Sections were blocked for 1 h at room temperature in 1 × PBS with normal goat serum. Sections were incubated in primary antibody (rabbit anti-c-fos, Synaptic systems, 226,003 or 226,008; 1:1,000 in blocking solution; rabbit anti-HA, Sigma-Aldrich, H6908, 1:2000 in blocking solution; Mouse anti-GluN1, Synaptic Systems, 114 011; 1:500 in blocking solution) at 4°C for 16 h. After incubation, sections were rinsed three times for 5 min in 1 × PBS. Sections were incubated in secondary antibody (ThermoFisher, goat anti-rabbit 488, 1:500; Thermofisher, goat anti-mouse 647, 1:500) for 2 h at room temperature followed by a three-time rinse with 1 × PBS, with a second rinse for DAPI staining. Sections were mounted in VECTASHIELD mounting medium (Vector Laboratories). Processing of confocal images was performed using Imaris (Bitplane, Zurich, Switzerland) software.

### Quantification and statistical analysis

Statistical analyses were performed using Prism 10 (GraphPad). Datasets that passed the normality test were compared through a two-tailed unpaired *t*-test. Comparison of non-normal datasets was tested through a two-tailed Mann–Whitney test. For one-sample comparisons in Fig. 1H and I, Supplementary Fig. 2B, One-sample Wilcoxon test was used. For Fig. 1E and Supplementary Fig. 2D, two-way ANOVA was used to measure the group effect. The statistical test used, exact value of the sample size, and statistical significance are reported in each figure legend.

## Supplementary Information

Additional file 1: Schematics and electrophysiological validation of NMDAR KD strategies. **A** Schematics for NMDAR KD strategy in the mPFC and the BLA using AAV-SaCas9. **B** Schematics for NMDAR KD strategy in the hippocampal CA1 using AAV-SaCas9. **C** Left, sgGrin1 group showed a significantly decreased level of NMDA/AMPA ratio compared to the control group. Each dot represents a cell; grey, control group injected with scrambled sgRNA; orange, test group injected with sgGrin1. Right, representative EPSC trace of each group. Unpaired t-test, ***p= 0.001. **D** Schematics for NMDAR KD strategy in the hippocampal CA1 using AAV-SaCas9 using transgenic LSL-Cas9-EGFP mice. **E** Same for **B** except that recording slices were obtained from LSL-Cas9-EGFP mice injected with AAV-CaMKII-Cre-U6-sgGrin1; black, uninfected control cells; red, injected cells identified with EGFP fluorescence. Right, representative EPSC trace of each group. Unpaired t-test, ****p< 0.0001. 

Additional file 2; Immunohistochemical analysis of expression level of GluN1 and Cas9 proteins. **A** Representative images of colocalization analysis of remote recall-tagged tdT+ ensemble and SaCas9+ ensemble. **B** Colocalization ratio of the tdT+ and the SaCas9+ ensembles. Each dot represents the average value of images from an individual mouse. Left, HA-labeled Cas9+ cells within the BLA and the mPFC. N= 8. Right, colocalization ratio normalized to the chance level, chance level = P(tdT+|DAPI) x P(Cas9+|DAPI). One-sample Wilcoxon test, mPFC, ##p= 0.0078; BLA, ##p= 0.0078. **C** Representative images of GluN1 immunohistochemistry. **D** Left, normalized intensity of fluorescence stained against GluN1 in the tdT+ and tdT- neurons. Two-way ANOVA followed by Šídák's multiple comparisons test. sgGrin1 ATG group, tdT- group, n= 135; tdT+ group, n= 101 from 3 mice; sgGrin1 group, tdT- group, n= 105; tdT+ group, n= 97 from 3 mice; Two-way ANOVA, tdT effect, ****p< 0.0001, Grin1 KD effect, ****p< 0.0001, interaction, ****p< 0.0001; Šídák's multiple comparisons test, sgGrin1 ATG group, ns, adjusted p> 0.9999, sgGrin1 group, ****p< 0.0001; Right, The GluN1 immunofluorescence of the GluN1+ tdT- cells. Mann-Whitney test, ns, p= 0.2210.

## Data Availability

The datasets used and/or analyzed during the current study are available from the corresponding author upon reasonable request.

## Abbreviations

*AFC*: Auditory fear conditioning
*CS*: Conditioned stimulus
*US*: Unconditioned stimulus
*NMDA*: N-methyl-d-aspartate
*AMPA*: α-Amino- 3-hydroxy- 5-methyl- 4-isoxazolepropionic acid
*mPFC*: Medial prefrontal cortex
*IL*: Infralimbic cortex
*BLA*: Basolateral amygdala
*AAV*: Adeno-associated virus
*DAPI*: 4,6 Diamidino- 2-phenylindole
*KD*: Knockdown
*CRISPR*: Clustered regularly interspaced short palindromic repeats
*SaCas9*: Cas9 from *Staphylococcus aureus*
*SaCas9*: Cas9 from *Staphylococcus pyogenes*
*sgRNA*: Single guide RNA
*4-OHT*: 4-Hydroxytamoxifen
*IHC*: Immunohistochemistry
*EPSC*: Excitatory postsynaptic current
*EPSP*: Excitatory postsynaptic potential
