# Supplementary figures and images for: Targeted NMDA receptor knockdown in recall‐activated neuronal ensembles impairs remote fear extinction
Source: Mol Brain. 2025 Apr 5;18:30. doi: 10.1186/s13041-025-01203-z (PMC11972453; doi:10.1186/s13041-025-01203-z)

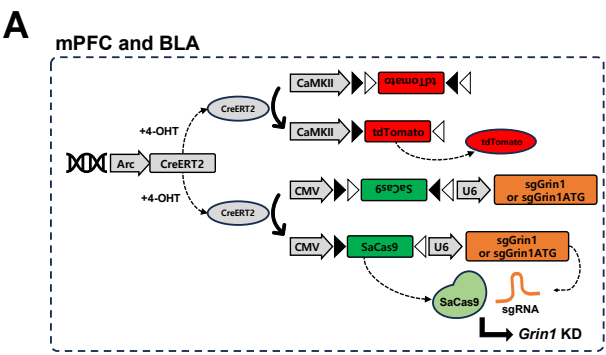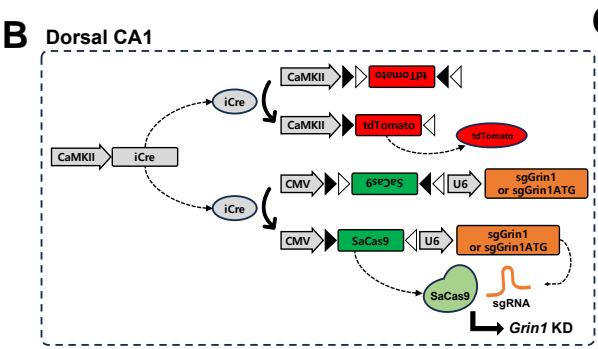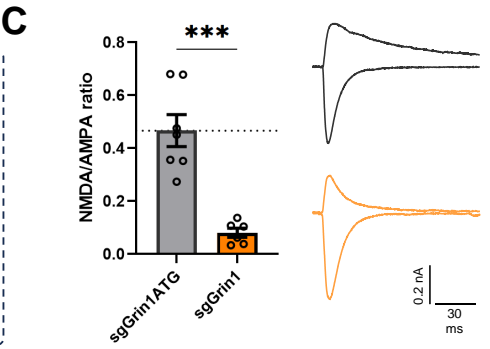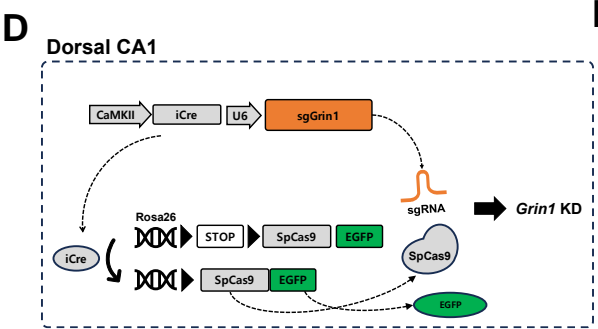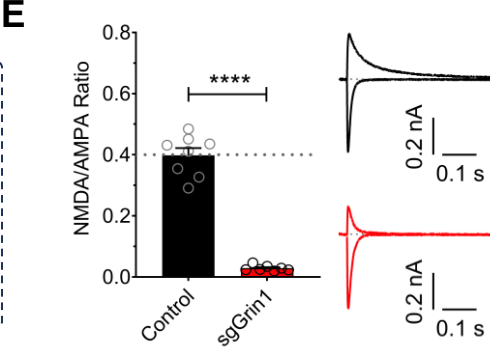

Supplement: Supplementary file 1 — Additional file 1: Schematics and electrophysiological validation of NMDAR KD strategies. A Schematics for NMDAR KD strategy in the mPFC and the BLA using AAV-SaCas9. B Schematics for NMDAR KD strategy in the hippocampal CA1 using AAV-SaCas9. C Left, sgGrin1 group showed a significantly decreased level of NMDA/AMPA ratio compared to the control group. Each dot represents a cell; grey, control group injected with scrambled sgRNA; orange, test group injected with sgGrin1. Right, representative EPSC trace of each group. Unpaired t-test, ***p= 0.001. D Schematics for NMDAR KD strategy in the hippocampal CA1 using AAV-SaCas9 using transgenic LSL-Cas9-EGFP mice. E Same for B except that recording slices were obtained from LSL-Cas9-EGFP mice injected with AAV-CaMKII-Cre-U6-sgGrin1; black, uninfected control cells; red, injected cells identified with EGFP fluorescence. Right, representative EPSC trace of each group. Unpaired t-test, ****p< 0.0001. [file 13041_2025_1203_MOESM1_ESM.pdf]

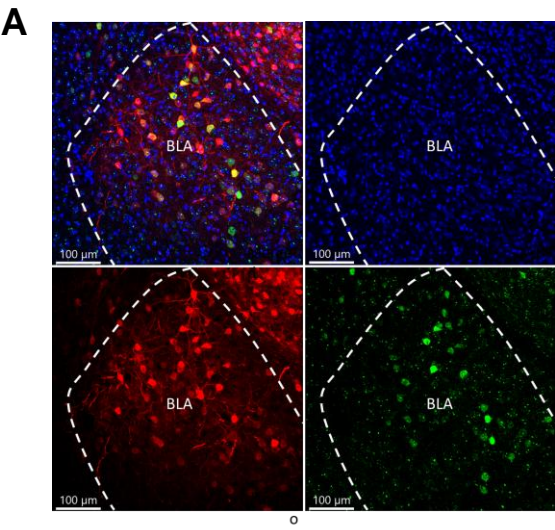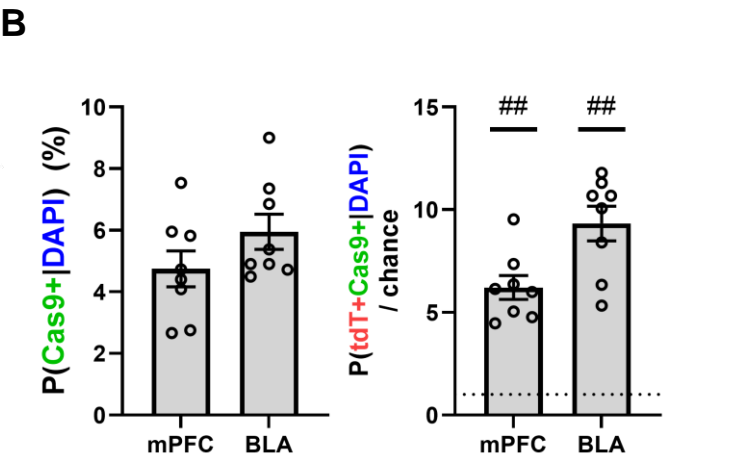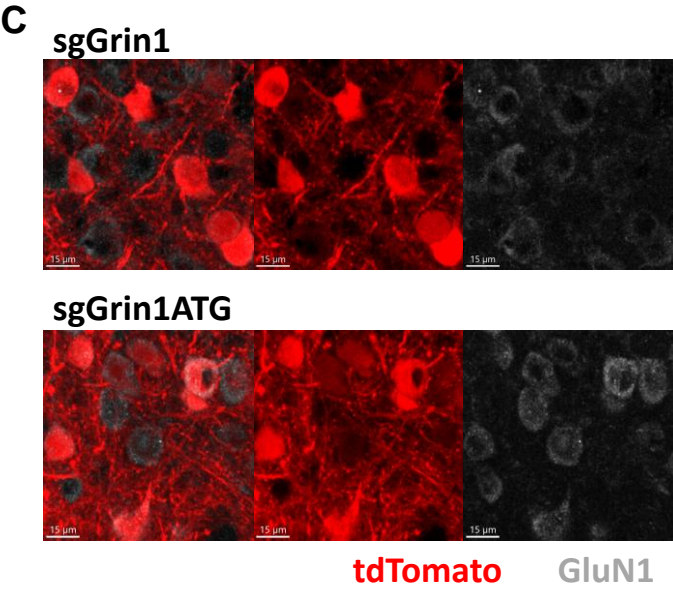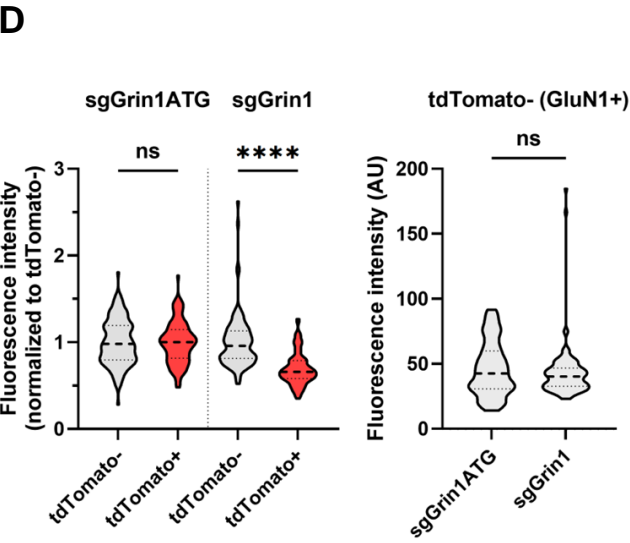

Supplement: Supplementary file 2 — Additional file 2; Immunohistochemical analysis of expression level of GluN1 and Cas9 proteins. A Representative images of colocalization analysis of remote recall-tagged tdT+ ensemble and SaCas9+ ensemble. B Colocalization ratio of the tdT+ and the SaCas9+ ensembles. Each dot represents the average value of images from an individual mouse. Left, HA-labeled Cas9+ cells within the BLA and the mPFC. N= 8. Right, colocalization ratio normalized to the chance level, chance level = P(tdT+|DAPI) x P(Cas9+|DAPI). One-sample Wilcoxon test, mPFC, ##p= 0.0078; BLA, ##p= 0.0078. C Representative images of GluN1 immunohistochemistry. D Left, normalized intensity of fluorescence stained against GluN1 in the tdT+ and tdT- neurons. Two-way ANOVA followed by Šídák's multiple comparisons test. sgGrin1 ATG group, tdT- group, n= 135; tdT+ group, n= 101 from 3 mice; sgGrin1 group, tdT- group, n= 105; tdT+ group, n= 97 from 3 mice; Two-way ANOVA, tdT effect, ****p< 0.0001, Grin1 KD effect, ****p< 0.0001, interaction, ****p< 0.0001; Šídák's multiple comparisons test, sgGrin1 ATG group, ns, adjusted p> 0.9999, sgGrin1 group, ****p< 0.0001; Right, The GluN1 immunofluorescence of the GluN1+ tdT- cells. Mann-Whitney test, ns, p= 0.2210. [file 13041_2025_1203_MOESM2_ESM.pdf]
